# Physicochemical Characterization, Antioxidant Capacity, and Sensory Properties of Murici (*Byrsonima* *crassifolia* (L.) Kunth) and Taperebá (*Spondias* *mombin* L.) Beverages

**DOI:** 10.3390/molecules26020332

**Published:** 2021-01-11

**Authors:** Adriana Aniceto, Julia Montenegro, Rafael da Silva Cadena, Anderson Junger Teodoro

**Affiliations:** 1 Nutrition Biochemistry Core, Laboratory of Functional Foods, Federal University of State of Rio de Janeiro, Av. Pasteur, 296, Rio de Janeiro 22290-240, Brazil; adriana.aniceto@edu.unirio.br (A.A.); juliamontenegro95@gmail.com (J.M.); rafaelcadena@gmail.com (R.d.S.C.); 2 Departament of Fundamental Nutrition, Federal University of State of Rio de Janeiro, Av. Pasteur, 296, Rio de Janeiro 22290-240, Brazil

**Keywords:** Amazonian fruits, experimental design, beverages, functional foods

## Abstract

Amazonian fruits are excellent sources of bioactive compounds and can be used in beverages to improve the nutritional and sensorial characteristics. The present study aimed to develop a blend of murici (*Byrsonima Crassifolia* (L.) Kunth) and taperebá (*Spondias Mombin* L.) through experimental design and investigating the nutritional and sensorial characteristics of fruits and beverages. The murici was highlighted as higher vitamin C content (58.88 mg · 100 g^−1^) compared to taperebá (25.93 mg · 100 g^−1^). The murici and taperebá are good sources of total phenolic compounds (taperebá 1304.15 ± 19.14 mgGAE · 100 g^−1^ and the murici of 307.52 ± 19.73 mg GAE · 100 g^−1^) and flavonoids (174.87 ± 1.76 μgQE/g and 129.46 ± 10.68 μgQE/g, murici and taperebá, respectively), when compared to other Brazilian fruits. The antioxidant capacity in different methods revealed that the taperebá had a higher average in the results, only in the ORAC method and did not present a significant difference (*p* > 0.05) in relation to the murici. The beverage development was performed using experimental design 2^3^, showed through sensory analysis and surface response methodology that murici and high sugar content (between 12.5 and 14.2% of sugar) influenced in sensory acceptance. Our findings indicate that beverages with improved nutrition and a sensory acceptance can be prepared using taperebá and murici fruits.

## 1. Introduction

The Amazon region has a rich biodiversity of fruit species, with approximately 220 species of edible fruit, representing 44% of the native fruit biodiversity in Brazil [1]. Fruit production in the Amazon represents only 0.2% of total production. The cultivation is mainly of exotic fruits that are consumed fresh or processed and contains a potential interest due to the increasing recognition of its nutritional and therapeutic value. [2,3,4]. The consumption of tropical fruit is increasing in local and international markets, where consumers lay emphasis on exotic character and the presence of potential nutrients capable of preventing some diseases [3].

Murici (*Byrsonima crassifolia* and *Verbascifolia* species) is small and round shape, between 1.7 and 2.0 cm in diameter, and weight between 4.45 and 5.45 g. The fruit is predominantly yellow, and the pulp is fleshy, soft, and juicy. A strong and unusual cheese flavor contributes to its unique and exotic flavor [5,6]. Approximately 19 polyphenolic compounds have been identified in murici, including gallotannins, quinic acid, gallates, proanthocyanins, quercetin derivates, and galloyl derivates, which are rarely found in fruits [7,8]. The fruit is considered to be a good source of carotenoids, and it contains more β-carotene than lycopene [9]. It also contains considerable amounts of lutein and zeaxanthin, but lesser than found in green leafy vegetables such as kale [10,11].

Taperebá is from *Spondia* genus, the fruits are round, elliptical, or ovoid-shaped drupes and the color of the peel and pulp is predominantly yellow. It has a fibrous and smooth texture and tastes sour-sweet and refreshing. [12]. They are rich in carotenoids, lutein is present in amounts higher than the zeaxanthins, and contain other types of carotenoids such as zeinoxanthin, β-cryptoxanthin, α-carotene, and β-carotene. It can be considered a source of pro-vitamin A, for example, a portion of 100 g provides about 37% of the RDI for adults [13].

The processing of pulps and fruit juices is an important agro-industrial activity, that adds economic value to the fruit, avoiding waste and minimizing losses that may occur during the marketing of the product *in natura*, in addition to being an alternative income to the fruits producer [14]. Fruit juices are becoming more popular due to their nutritional and functional properties; however, 100% tropical juices are not accepted by some consumers, due to the exotic flavor. [15]. In order, to minimize this kind of rejection by consumers, more and more industries have been developing products using blends. The blends exhibit numerous advantages such as the possibility of combining different flavors, taste and the sum of nutritional components not found in individual juices and nectars [16].

During the formulation of functional products, it is essential to assess from the first stage of development how the product would meet the nutritional demands of consumers, in addition to providing acceptance and adequate sensory properties [17]. Optimization techniques, when used in the correct way, can be a helpful tool, to ensure optimal composition and promote understanding of the sensory characteristics by consumers preferences, obtaining a formulation that allows maximum acceptance [18,19].

In this context, combining the nutritional characteristics with the sensory and functional benefits, murici and taperebá appears to be a promising alternative in the development of new functional products. Therefore, the aim of the study was a development of a beverage blend of murici and taperebá, using the sensory attributes and response surface methodology for beverage (functional and sensory) characteristics optimization.

## 2. Results and Discussion

### 2.1. Physicochemical Analysis of Taperebá and Murici Pulp

The physicochemical analysis, bioactive compounds, and antioxidant activity of the murici and taperebá pulps can be observed in Table 1.

The results showed that taperebá presented higher values of acidity when compared to murici. In a similar study, authors obtained, for murici, values of 0.77g · 100 g^−1^ of citric acid for acidity and pH 3.93 [20]. In another study, the analysis of taperebá showed values of 1.86 g · 100 g^−1^ of citric acid for acidity and pH = 2.53 [21], similar to those found in our study. Acidity is one of the criteria that affect the classification of fruit-based flavor. Fruits with acidity levels ranging from 0.08 to 1.95% can be classified as mild in flavor and are well accepted for consumption as fresh fruit [22].

In relation to the total soluble solids content, taperebá presented a higher content of soluble solids compared to the murici. Studies conducted previously demonstrated 10.09 °Brix for taperebá [21] and 5.20 °Brix for murici [23]. The total soluble solids content (TSSC) shows high positive correlation with sugars content, and therefore is generally accepted as an important quality trait of fruits [24].

Murici was identified with a higher vitamin C content compared to taperebá. An amount of 92.59 mg · 100 g^−1^ of vitamin C in murici was observed [23], with this value being above that found in our study, and this difference can be justified by considering the light and oxygen sensitivity of Vitamin C. The study carried out obtained 23.72 mg · 100 g^−1^ of vitamin C in taperebá [21]. This vitamin contains a significant antioxidant, primarily derived from fruits, and supports the body. High correlation between antioxidant activity using DPPH, FRAP, and ORAC assays and vitamin C was likely to be found only in fruits that contain high vitamin C such as citrus fruits [25].

### 2.2. Total Phenolic and Flavonoids Content of Taperebá and Murici Pulp

The highest amount of total phenolic content was found in the taperebá when compared to murici. A study carried out in 2013 obtained 222.2 ± 6.1 mg GAE · 100 g^−1^ for murici. In the same study, the amount of phenolic compounds of *guapeava* (*Pouteria guardneriana* Radlk) and *gabiroba* (*Campomanesia cambessedeana* Berg) were higher than in murici, with 851.0 ± 40.7 mg GAE · 100 g^−1^ and 321.1 ± 5.6 mg GAE · 100 g^−1^, respectively [4]. Another study indicated in traditional fruits such as pineapple (*Ananas comosus* (L.) Merran total phenolic content of 38.1 ± 0.7 mg GAE · 100 g^−1^ and papaya (*Carica papaya* L.) 53.2 ± 3.6 mg GAE · 100 g^−1^ [26]. The differences may be related to several factors that can influence the levels of bioactive compounds in exotic and traditional fruits such as type of cultivation, climate, variety of fruits, and time of year.

Flavonoid content determined by a spectrophotometric method showed that murici presented higher amounts of flavonoids (174.87 ± 1.76 µg QE/g) compared to the taperebá (129.46 ± 10.68 QE/g), unlike that observed with total phenolic content. Similarly to our study, murici also had higher levels of flavonoids than taperebá, of which values were 13.8 mg/100 g for murici and 7.1 mg/100 g for taperebá [3].

### 2.3. Antioxidant Activity of Murici and Taperebá Pulp

According to the DPPH test, the highest percentage of reduction was observed in taperebá. A reduction (%) of the DPPH radical around 55.97 ± 3.20% was reported in the study for taperebá [27], a significant difference (*p* < 0.05) when compared with the result obtained in our study and very similar to the murici values.

In the FRAP method, the result obtained for the taperebá pulp was higher compared to the murici pulp. Another study verified an amount of 11.8 μmol of Fe_2_SO_4_/g in taperebá using the same methodology. In the same study, it was observed that *camu-camu* (*Myrciara dubia*), another Amazonian fruit, obtained a value of 279 μmol Fe_2_SO_4_/g, with the fruit having greater antioxidant capacity among the fruits studied [3].

Observing the TEAC assay, there was no significant difference (*p* > 0.05) in the antioxidant capacity values between the different extracts for the murici. For the taperebá, acetone 70% obtained the highest amount of extraction with values of 188.24 ± 65.46 μmol trolox/g.

Evaluating the results obtained of antioxidant capacity considering the ORAC test, there was no significant difference (*p* > 0.05) between the murici and taperebá pulp. A study observed, for the ORAC assay, that murici presented an amount of 3352.3 ± 952.0 μmol TE/100 g [4]. The potent antioxidant activity of murici and taperebá fruit pulp may be associated with their content of vitamin C and phenolic compounds, as well as with the occurrence of carotenoids in their composition [28].

### 2.4. Physicochemical Analysis of Beverages

The physicochemical analysis of the beverages samples is shown in Table 2. It was observed that the pH values of the beverages presented small variation with maximum and minimum values of 3.20 and 2.87. Samples 4 and 13 presented a statistical difference (*p* < 0.05) from the other formulas. Similar values were observed in other study, with nectars blended with taperebá (mango and taperebá) obtaining a pH value of 3.15 [5]. Another study obtained a value of 3.3 in the pineapple and murici blend [16].

The maximum values of acidity were observed in 5 and 14 samples, 0.35 and 0.36 g citric acid · 100 g^−1^, respectively, with statistical difference for the other samples (*p* < 0.05). The values are different from the samples developed in other study, whose observed results were 1.88 g citric acid · 100 g^−1^ for passionfruit and murici blend and 2.20 g citric acid · 100 g^−1^ for the passionfruit and taperebá blend [16]. The difference can be explained considering that the mixtures developed in the comparative study contain a higher amount of pulp, do not contain added sugar, and the passionfruit is characterized by being a very acid fruit.

Regarding the values obtained of soluble solids, there was a significant difference between the beverages developed (*p* < 0.05). The lowest value was observed in sample 15 (8.0 °Brix), characterized by the lower amount of sugar added, and the highest value was in sample 16 (16.47 °Brix), characterized by the higher amount of sugar added. The value of sample 16 is similar to that observed in another study, of mango and taperebá blend (15 °Brix) [5].

For vitamin C, the values are between 12.96 and 19.25 mg · 100 g^−1^. The value of 19.25 mg · 100 g^−1^ can be justified by the high pulp concentration in the sample (195 g/L for each pulp). The values identified in another study for pineapple and taperebá blend and pineapple and murici blend were 27.44 and 31.12 mg · 100 g^−1^, respectively, and in the passionfruit and taperebá blend and passionfruit and murici blend were 29.23 and 34.20 mg · 100 g^−1^, respectively [16]. The interactions of bioactive compounds (additive, synergistic, or antagonistic) are not well explored in situations of fruit combination (for example, beverages based on Amazonian fruits). In addition, the fruits used in this study is native and under-exploited and ‘in vitro’ properties of a product obtained by a combination of murici and taperebá were not explored.

Another study of bioactive compounds and antioxidant activity in exotic fruits observed that the antioxidant activities in the analyzed fruits cannot be attributed only to their phenolic compounds, but also to the actions of different antioxidant compounds present in the fruits and in possible synergistic and antagonistic effects still unknown [26].

### 2.5. Sensory Analysis

The acceptance sensory results of murici and taperebá blend beverages for the attributes overall liking, flavor, taste, texture and appearance are presented in Table 3. In general, all samples obtained mean values higher than 6.0 with exception for samples 3 and 15. Thus, it can demonstrate that the samples in all attributes evaluated had good acceptance.

The beverages had higher mean values for appearance attribute in comparison to the other attributes, with mean values between 6 (like slightly) and 7 (liked moderately), except for samples 3 and 4. The lowest mean 6.34 in attribute appearance was from sample 4. Observing the characteristics, this sample contained less taperebá pulp compared to the other samples (74.4 g/L). This characteristic may have influenced the low appearance acceptance of the sample, since the taperebá pulp presents an intense yellow color, which directly affects the appearance of the beverage.

It was observed in a sensory study that beverages with the most intense yellow color were considered the most attractive to the consumer, thus increasing their acceptance. The study evaluated a beverage containing passionfruit juice, where it was expected that the samples with color and aroma more characteristic of the fruit would have greater acceptance by the consumers. This confirms the higher levels of acceptance for beverages with a stronger yellow color [17].

In relation to the flavor attribute, samples 10, 13, 14, 16, and 17 obtained the highest mean values between 6.71 and 6.83 and did not present significant differences between them (*p* > 0.05).

The sample 10 presented the highest mean value for all attributes, with means between 6.73 and 7.28, in comparison to the other samples. The sample contains 10% sugar, 150 g/L of taperebá, and 74.4 g/L of murici, being the beverage with the least murici pulp content among the samples evaluated.

The lowest mean value of the taste attribute was observed in formulation 15, with a mean value of 5.09. Sample 15 contains the lowest sugar concentration (5.8%) among the samples suggesting that the lower the sugar content, the lower the acceptance of the product.

A study showed that the concentration of sugar in the blend formulation of soy and grapes in the proportion 1:1.5 and 1:2 interfered in the acceptance by the consumers for the taste attributes and overall liking, being that the beverages with the highest sugar concentration (14 °Brix) were the most accepted. This behavior demonstrates the preference of consumers for more sweet beverages [29].

### 2.6. Beverage Optimization

Table 4 shows the ANOVA test with the respective *p* values in relation to the sensory attributes of aroma, taste, appearance, texture, and overall liking. Analyzing the *p* values for the aroma attribute, it can be observed that only the sugar constituent showed a significant difference (*p* = 0.0051). This means that the variation of the other constituents (murici and taperebá) did not influence the acceptance result in relation to this attribute.

Table 4 showed that the three constituents (taperebá, murici, and sugar) has influenced the appearance of all evaluated attributes (*p* < 0.05). The taperebá constituent also significantly influenced the texture attribute, indicating that the variations of taperebá concentration does not influence, mainly, the attribute taste and overall liking acceptance.

It can be observed in Table 4 that the sugar constituent presented the lowest *p* values in relation to the constituents taperebá and murici, strongly indicating the high influence in relation to the variation of the amounts added in the beverage.

**Figure 1:**
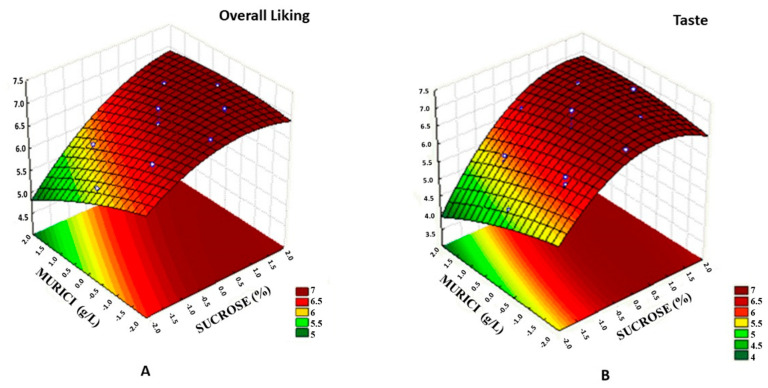
Response surface as a function of the murici pulp concentration and sugar concentration, with a concentration of 150 g/L of fixed taperebá pulp. (**A**) Overall liking attribute and (**B**) taste attribute.

Regarding the acceptance of the taste and overall liking attributes, was reported significant effects (*p* < 0.05) for sugar (*p* = 0.0000 and *p* = 0.0001) and murici (*p* = 0.0260 and *p* = 0.0056) were reported. The choice of concentrations can be explained from the response surface graphs (Figure 1) for the overall liking and flavor attributes in murici and taperebá beverages. It was observed that the highest acceptance means are reached with the highest sugar concentration.

Figure 1A revealed that the highest acceptance mean of the overall liking attribute was comprised in a concentration of murici pulp between 105 and 195 g/L and between 12.5 and 14.2% of sugar, indicating a direct relationship between increased sugar concentration and mean acceptance. The same results can be observed in Figure 1B, where the highest acceptance mean of the taste attribute was comprised in a concentration of murici pulp between 105 and 195 g/L and between 12.5 and 14.2% of sugar. The response surface of the overall liking, taste, texture, and appearance attributes can be seen in Figure S1. It was observed that for texture, the smaller amount of murici the greater acceptance, and for appearance, the amount of taperebá influences the acceptance of this attribute. From these results, bioactive compounds and antioxidant capacity analyses of the three beverages in the optimized region were performed.

### 2.7. Antioxidant Capacity of Optimized Beverages

The optimized samples were determined considering the acceptance of the attribute overall liking and taste, setting the amount of taperebá pulp at 150 g/L and varying the sugar amounts between 12.5 and 14.2% and murici between 105 and 195 g/L. Table 5 shows the quantities of the independent variables in the optimized samples.

**Figure 2:**
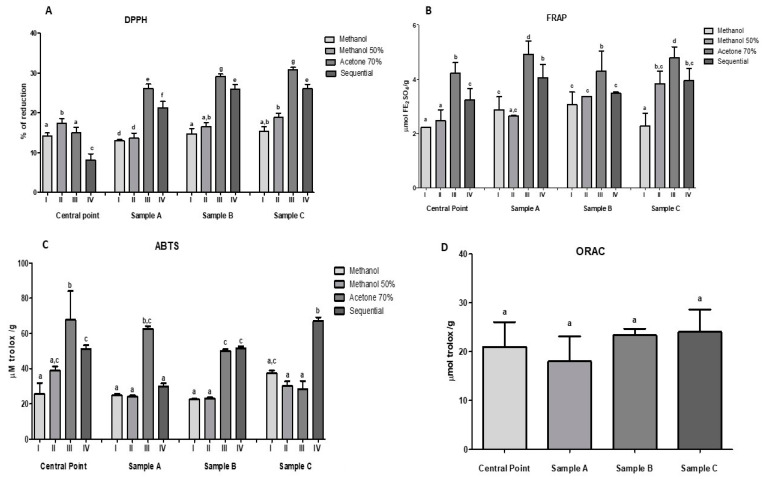
Antioxidant capacity of the optimized formulas and central point. (**A**) DPPH method, (**B**) FRAP Method, (**C**) ABTS Method and (**D**) Oxygen Radical Absorbance Capacity (ORAC) Test. Different letters indicate significant difference (*p* < 0.05).

For antioxidant capacity, the following were considered: DPPH, FRAP ABTS, and ORAC methods, using four different extractive agents: Methanol (I), methanol 50% (II), acetone 70% (III), and Sequential (IV). Figure 2 shows the antioxidant capacity of optimized samples

In the DPPH method, sample C obtained the highest value with 30.84 ± 0.53% of reduction, whereas the center point obtained the lowest reduction with 8.10 ± 1.60%. These results can be explained because sample C contains the highest amount of fruit pulps (345 g/L of mixture of murici and taperebá. No statistical differences (*p* > 0.05) were observed between samples A and C by FRAP assay, except when using methanol as an extraction solution.

Considering the ABTS method, it is observed that there is no significant difference (*p* > 0.05) between the center point (Acetone 70%) and sample C (Sequential), with values between 67.68 ± 16.37 and 67.04 ± 0.20 μmol trolox/g, respectively. For the ORAC test, it has been demonstrated that there was no significant difference between the optimized samples and the central point. The sample C obtained the highest value 24.05 ± 4.54 μmol trolox/g, being the sample with the highest fruit pulp content (345 g/L of the murici and taperebá mixture). The total antioxidant capacity for tropical fruits juice, using the ABTS and ORAC assays, were conducted in other study. The results obtained were 167.17 ± 4.10 and 235.90 ± 11.90 mMTrolox/g in the lyophilized product, respectively. The fruit blend optimized used as comparison to our study is composed of 10% *acerola (M. punicifolia*), 5% *acai (E. oleracea*), 5% yellow mombin (*Spondias lutea* L.), 5% cashew apple (*A. occidentale* L.), 5% *camu-camu (M. dubia*), 20% pineapple (*Ananas comosus* L.), 50% water and adjusted with sucrose to 12 °Brix of soluble solids [30].The difference in the results obtained may be related to the composition, synergy between the fruits, and the method of analysis used. The comparative study used a composition with six fruits, while in our study, the beverage was made with two fruits

## 3. Materials and Methods

### 3.1. Material and Samples

Samples of commercial frozen fruit pulps of murici (*Byrsonima crassifolia* (L.) Kunth) and taperebá (*Spondias mombin* L.) were obtained from producers in the State of Pará and all samples were kept frozen in freezers in the original packaging at −18 °C until the preparation of the beverages. The sucrose was purchased at local market. All the solvents and standards used for the antioxidant assays and phenolics content were also purchased from Sigma-Aldrich (Sigma-Aldrich, St. Louis, MO, USA).

### 3.2. Physicochemical Characterization

All physicochemical analyses were performed in triplicate according to the AOAC methodology (AOAC, Association of official analytical chemists, 2000, 1995). The pH was determined using a pH-meter (Tecnopon, Piracicaba, Brazil) and it was expressed as the negative logarithm of the hydrogen ion concentration in a solution. Total soluble solids were determined through refraction index with a refractometer at 20 °C and the results were expressed as °Brix. Titratable acidity was determined using titration with NaOH 0.1 N, the results were expressed as g · 100 g^−1^ of citric acid per 100 g of sample. Vitamin C content was determined by a titration method, using the reduction of 2,6-dichlorophenolindophenol (DCFI) indicator by the ascorbic acid, and the results were expressed in mg of ascorbic acid per 100 g of sample.

### 3.3. Extraction

First, 2.5 g of the samples were added in 10mL of the extractor solutions: (I) Methanol, (II) methanol 50%, (III) acetone 70%, and (IV) sequential, remained in agitation in ultraturrax for 2 min and were submitted to centrifugation (Heraeus Multifuge X3FRCentrifuge Thermo Fisher Scientific, Waltham, MA, USA) for 15 min. The supernatant was transferred to a 50 mL volumetric flask and filled with distilled water. The extract obtained was used for total phenolic content, antioxidant capacity analysis: 2,2-diphenyl-1-picrylhydrazyl (DPPH), 22,2′-azino-bis(3-ethylbenzothiazoline-6-sulfonic acid) (TEAC), and Ferric reducing antioxidant power (FRAP) assay.

For flavonoids analysis, the extract was obtained whereby 10 g of the sample was added to 10 mL of methanol, stayed in agitation in ultraturrax for 2 min, and was centrifuged for 15 min. The supernatant was transferred to a 25 mL volumetric flask and filled with distilled water.

### 3.4. Determination of the Total Phenolic Content

Total phenolic content was determined by the Folin–Ciocalteau method [31]. Extracts (1.0 mL) were added with 2.5 mL of Folin–Ciocalteau reagent and 2 mL of 4% sodium carbonate solution. The solution was incubated at dark ambient for 2 h. The absorbance was measured at 760 nm in the spectrophotometer (Shimadzu UV-2700, Shimadzu Corporation, nakagyo-ku kyoto, Japan) and the results were expressed as gram of gallic acid equivalents (GAE) per 100 grams of sample using a gallic acid (2.5–50 μg/μL) standard curve.

### 3.5. Determination of the Flavonoids Content

The flavonoid content was determined using the methodology adapted from [32]. 2 mL of extract, 1 mL of 5% (*w/v*) aluminum chloride in methanol, and 2 mL of methanol were mixture and a solution prepared. The solution reacted during 30 min in the dark and the reading was taken at 420 nm using a spectrophotometer (Shimadzu UV-2700). Quercetin was used to make a standard curve and the flavonoid content is expressed in µg of quercetin equivalent per gram of the sample.

### 3.6. TEAC Assay

TEAC assay was performed following the procedure proposed by Thaipong et al. [33]. TEAC assay (22,2′-azino-bis(3-ethylbenzothiazoline-6-sulfonic acid)) cations were produced by reacting potassium persulfate. The ABTS^+^ solution was diluted with ethanol to an absorbance of 0.70 ± 0.02 at 734 nm using a spectrophotometer (Shimadzu UV-2700). Aliquots of volumes of samples (10, 25, and 50 µL) were used to subtract a final volume of 3 mL in each reading. Trolox (6-hydroxy-2,5,7,8-tetramethylchroman-2-carboxylic acid) was used as a standard curve. Results were expressed in µmol Trolox equivalent per gram of sample.

### 3.7. DPPH (Free Radical-Scavenging) Assay

The antioxidant capacity using DPPH assay was determined according to the method based on the quantification of free radical-scavenging [34]. The samples were added to react with stable radical DPPH (2,2-diphenyl-1-picrylhydrazyl) in methanol solution. The reduction of DPPH radical was measured by reading the absorbance at 515 nm using a spectrophotometer (Shimadzu UV-2700) at 30 min of reaction. The antioxidant capacity was expressed as the concentration of antioxidant required to reduce the original amount of free radicals by 50% (EC50) and values were expressed % of reduction.

### 3.8. FRAP Assay

The FRAP assay was done according to Abreu et al. [35]. The FRAP reagent was prepared using 25 mL of 0.3 M acetate buffer (pH 3.6), 2.5 mL of 10 mM TPTZ (2,4,6-tripyridyl-s-triazine) solution, and 2.5 mL of 20 mM ferric chloride solution. 2.7 mL of FRAP reagent at 37 °C was added with 90 µL of sample extract and 270 µL of distilled water. After 30 min in warm bath, the readings of samples were taken at 595 nm using a spectrophotometer (Shimadzu UV-2700). The ferrous sulfate was used to the standard curve and the results were expressed µM ferrous sulfate per gram of sample.

### 3.9. ORAC (Oxygen Radical Absorbance Capacity) Assay

The ORAC assay was determined using an automated plate reader (SpectraMax i3x, Molecular Devices, USA) with 96-well plates [36]. Analyses were conducted in phosphate buffer pH 7.4 at 37 °C. A peroxyl radical was generated using (2,2’-azinobis (2-amidinopropane), prepared for each run. Fluorescein was used as the substrate. Fluorescence conditions were as follows: Excitation at 485 nm and emission at 535 nm. The standard curve was generated, and the results were expressed in µmols Trolox equivalents per gram of sample.

### 3.10. Experimental Design

The beverages were developed according to factorial design 2^3^ (Table 6), using the following independent variables: Concentration of taperebá pulp in g/kg (X_1_), concentration of murici pulp in g/kg (X_2_), sugar concentration in % (X_3_). The dependent variables were the sensory attributes: Appearance, aroma, taste, texture, and overall liking.

The fruit pulp content used in experimental design was determined in accordance with Brazilian legislation (Brazil, 2003) establishing a minimum amount of 30% of the mixture of two or more juices or fruit pulp for complying of Nectar Blend category.

The standard percentage (%) of sugar was based on the results presented in other study that determined the ideal sweetness for Amazonian fruit juices, and found that the optimum concentration of sugar to the *cupuaçu*, acerola and *açai* blend was 9.5 g/100 mL and 10.7 g/100 mL to the soursop, *camu-camu,* and taperebá blend [37]. For acceptance test, 17 beverages were produced, containing 6 axial points and 3 central points.

### 3.11. Beverage Preparation

The components of the formulation were weighed and mixed in suitable equipment until complete homogenenization. The beverages were packaged in sanitized plastic packaging with 2000 mL and stored under refrigeration at 7–10 °C until the moment of sensory tests. The preparation and storage of beverages were conducted at the Technical Laboratory and Dietetics at the Federal University of the State of Rio de Janeiro, following the Good Manufacturing Practices.

### 3.12. Sensory Analysis

The acceptance test was performed with 100 consumers chosen at random from different age and gender groups in Federal University of State of Rio de Janeiro. The evaluation was carried out in a sensory laboratory of Federal University of State of Rio de Janeiro using individual cabins under artificial lighting. The evaluation was conducted with 17 samples in three stages, in the first and second stages 6 samples were evaluated, and in the third stage 5 samples. The samples were served at a refrigerated temperature (7 °C) in a monadic way, in plastic cups coded with 3-digit random numbers, with approximately 30 mL. Measuring the sensory attributes of appearance, aroma, taste, texture, and overall liking, a 9-point hedonic scale was used (9 = like extremely; 1 = dislike extremely). The expressions were converted to numerical values and analyzed. The research protocol of the study was approved by the Ethics Committee of Federal University of State of Rio de Janeiro (number 39693914.8.0000.528).

### 3.13. Statistical Analysis

The experimental design was performed with Statistica^®^ v.8.0 (Statsoft). Analysis of variance (ANOVA) and Tukey test, with 95% reliability, were used for physicochemical and antioxidant capacity to compare the statistical differences. *p*-values lower than 0.05 were considered significant. The results were expressed as the mean ± standard deviation or just mean using the ASSISTAT, version 7.7 beta.

## 4. Conclusions

The murici and taperebá pulps are good sources of vitamin C, total phenolic compounds, and flavonoids, when compared to other Brazilian fruits. Taperebá had a higher antioxidant capacity in the DPPH, ABTS, and FRAP methods, and did not present a significant difference (*p* > 0.05) in the ORAC in relation to the murici. Thus, it can be considered that taperebá has higher antioxidant activity. Response surface methodology demonstrated that murici and taperebá blends are a well-accepted product with significant bioactive and antioxidant features. The combination of murici and taperebá can act synergistically in the improvement of the antioxidant capacity in the beverage’s development. Further studies considering the nutritional claims, physical properties, and texture/rheological parameters related to sensorial changes after the industrial processing, will help to understand the applicability of these products in the commercial functional beverages.

## Figures and Tables

**Table 1:** Physicochemical characterization, bioactive compounds, and antioxidant activity of murici and taperebá pulp.

Parameter	Murici	Taperebá
Acidity (g citric acid · 100 g^−1^)	0.75 ± 0.03 ^a^	1.74 ± 0.05 ^b^
Vitamin C (mg · 100 g^−1^)	58.88 ± 1.63 ^a^	25.93 ± 1.65 ^b^
Total Soluble Solids (°Brix)	4.20 ± 0.01 ^a^	9.80 ± 0.10 ^b^
pH	3.36 ± 0.01 ^a^	2.60 ± 0.01 ^b^
Total Phenolics Compounds (mg GAE. 100 g^−1^)	307.52 ± 19.73 ^a^	1340.15 ± 19.14 ^a^
Flavonoids (µg QE/g)	174.87 ± 1.76 ^a^	129.46 ± 10.68 ^b^
DPPH (% of reduction)	52.94 ± 2.41 ^a^	74.14 ± 1.34 ^b^
FRAP (µmol FE_2_SO_4_/g)	7.38 ± 0.98 ^a^	14.36 ± 3.47 ^b^
ABTS (μmol trolox/g)	79.49 ± 3.35 ^a^	188.24 ± 65.46 ^b^
ORAC (µmol trolox/g)	312.54 ± 82.95 ^a^	332.46 ± 86.82 ^a^

Equal letters in same line indicate that the values are not statistically significant (*p* ≤ 0.05) by Tukey test. GAE = Gallic acid equivalents; QE = Quercetin

**Table 2:** Murici and taperebá blend beverages physicochemical analysis results.

	Physicochemical Atributes (*)			
Samples	pH	Acidity	Soluble Solids	Vitamin C
		(g·100 g^−1^)	(°Brix)	(mg·100 g^−1^)
1	3.05 ± 0.01 ^bc^	0.18 ± 0.01 ^g^	8.80 ± 0.00 ^m^	17.57 ± 2.77 ^abc^
2	2.87 ± 0.01 ^h^	0.26 ± 0.01 ^de^	9.73 ± 0.23 ^j^	14.40 ± 4.16 ^abc^
3	3.00 ± 0.05 ^cde^	0.24 ± 0.00 ^e^	9.40 ± 0.00 ^l^	18.77 ± 0.69 ^ab^
4	3.20 ± 0.03 ^a^	0.19 ± 0.01 ^fg^	11.53 ± 0.12 ^h^	14.18 ± 2.36 ^abc^
5	2.94 ± 0.03^fg^	0.35 ± 0.01 ^a^	13.00 ± 0.00^f^	13.30 ± 2.95 ^bc^
6	3.00 ± 0.01 ^cde^	0.27 ± 0.00 ^de^	12.13 ± 0.12 ^g^	15.97 ± 0.69 ^abc^
7	3.02 ± 0.01 ^bcd^	0.31 ± 0.04 ^b^	10.20 ± 0.00 ^i^	17.77 ± 1.18 ^abc^
8	3.07 ± 0.01 ^b^	0.18 ± 0.00 ^fg^	14.00 ± 0.00 ^e^	13.75 ± 0.70 ^abc^
9	3.03 ± 0.01 ^bcd^	0.27 ± 0.00 ^de^	14.60 ± 0.00 ^c^	15.25 ± 0.70 ^abc^
10	2.99 ± 0.01 ^def^	0.21 ± 0.00 ^f^	12.00 ± 0.00 ^g^	19.10 ± 1.86 ^ab^
11	3.00 ± 0.01 ^cde^	0.31 ± 0.01 ^bc^	13.00 ± 0.00 ^f^	16.13 ± 1.85 ^abc^
12	3.00 ± 0.01 ^cde^	0.27 ± 0.01 ^cde^	12.03 ± 0.06 ^g^	16.77 ± 1.20 ^abc^
13	3.15 ± 0.01 ^a^	0.26 ± 0.01 ^de^	14.27 ± 0.12 ^d^	12.96 ± 1.40 ^c^
14	2.96 ± 0.01 ^ef^	0.36 ± 0.01 ^a^	15.47 ± 0.12 ^b^	19.25 ± 1.20 ^a^
15	3.02 ± 0.01 ^bcd^	0.27 ± 0.00 ^de^	8.00 ± 0.00 ^n^	14.63 ± 0.68 ^abc^
16	2.90 ± 0.02 ^gh^	0.27 ± 0.01 ^de^	16.47 ± 0.12 ^a^	13.69 ± 2.51 ^abc^
17	2.98 ± 0.03 ^def^	0.28 ± 0.01 ^bcd^	12.00 ± 0.00 ^g^	15.17 ± 0.69 ^abc^

(*) Mean ± SD. Different letters in the same column represent statistically different results according to the Tukey’s test (*p* < 0.05.)

**Table 3:** Murici and taperebá blend beverages sensory analysis results.

	Sensory Atributes (*)				
Samples	Aroma	Taste	Appearance	Texture	Overall Liking
1	6.09 ^abc^	6.06 ^bcd^	6.54 ^bc^	5.99 ^bcde^	6.30 ^bcdef^
2	6.31 ^abc^	6.26 ^abcd^	6.77 ^abc^	6.40 ^abcde^	6.58 ^abcde^
3	5.66 ^bc^	5.57 ^de^	6.36 ^c^	5.55 ^e^	5.78 ^f^
4	5.87 ^bc^	6.35 ^abcd^	6.34 ^c^	6.16 ^abcde^	6.38 ^abcdef^
5	6.21 ^abc^	6.35 ^abcd^	6.85 ^abc^	6.60 ^abcd^	6.59 ^abcde^
6	6.18 ^abc^	6.59 ^abc^	6.92 ^abc^	6.56 ^abcd^	6.68 ^abcd^
7	6.16 ^abc^	5.77 ^cde^	6.97 ^abc^	6.08 ^abcde^	6.17 ^def^
8	6.38 ^abc^	6.79 ^ab^	7.00 ^abc^	6.78 ^abc^	6.79 ^abcd^
9	6.64 ^ab^	6.99 ^a^	7.32 ^a^	6.94 ^a^	7.10 ^a^
10	6.73 ^a^	6.92 ^ab^	7.28 ^ab^	6.85 ^ab^	7.04 ^ab^
11	6.28 ^abc^	6.29 ^abcd^	6.64 ^abc^	5.84 ^de^	6.24 ^cdef^
12	6.38 ^abc^	6.57 ^abc^	7.05 ^abc^	6.20 ^abcde^	6.54 ^abcdef^
13	6.71 ^a^	6.94 ^ab^	6.69 ^abc^	6.56 ^abcd^	6.84 ^abcd^
14	6.83 ^a^	6.76 ^ab^	6.78 ^abc^	6.55 ^abcd^	6.86 ^abcd^
15	6.25 ^abc^	5.09 ^e^	6.82 ^abc^	5.93 ^cde^	5.87 ^ef^
16	6.77 ^a^	7.01 ^a^	6.97 ^abc^	6.79 ^abc^	6.98 ^abc^
17	6.78 ^a^	7.13 ^a^	6.96 ^abc^	6.70 ^abcd^	7.03 ^ab^

(*) Mean. Different letters in the same column represent statiscally different results according to the Tukey’s test (*p* < 0.05.)

**Table 4:** Values of *p* for sensorial acceptance for murici and taperebá blend beverage.

	*p* Values				
Constituents	Aroma	Taste	Appearance	Texture	Overall Liking
Taperebá (1)	0.0752	0.5944	0.0028	0.0290	0.0675
Taperebá quadratic	0.0616	0.0740	0.0089	0.5122	0.1461
Murici (2)	0.3420	0.0260	0.0048	0.0025	0.0056
Murici quadratic	0.7539	0.5594	0.8678	0.3881	0.6006
Sucrose (3)	0.0051	0.0000	0.0202	0.0004	0.0001
Sucrose quadratic	0.7342	0.0063	0.4605	0.4384	0.0789
1 and 2	0.8259	0.5309	0.6887	0.9249	0.7183
1 and 3	0.5964	0.5309	0.2702	0.1665	0.5010
2 and 3	0.1158	0.1625	0.0459	0.7779	0.1665

**Table 5:** Formula optimization uncoded and coded for murici and taperebá blend.

Samples	Taperebá	Murici	Sucrose
	(g/L)	(g/L)	(%)
A	150 (0)	105 (−1)	12.5 (+1)
B	150 (0)	150 (0)	14.2 (+1.68)
C	150 (0)	195 (+1)	12.5 (+1)
Central Point	150 (0)	150 (0)	10 (0)

**Table 6:** Factorial design 2^3^ for preparation of murici and taperebá beverages blends (uncoded and coded values).

	Uncoded And Coded Values		
Samples	Taperebá	Murici	Sucrose
	(g/L)	(g/L)	(%)
1	105 (−1)	105 (−1)	7.5 (−1)
2	195 (+1)	105 (−1)	7.5 (−1)
3	105 (−1)	195 (+1)	7.5 (−1)
4	74.4 (−1.68)	150 (0)	10 (0)
5	225.6 (+1.68)	150 (0)	10 (0)
6	150 (0)	150 (0)	10 (0)
7	195 (+1)	195 (+1)	7.5 (−1)
8	105 (−1)	105 (−1)	12.5 (+1)
9	195 (+1)	105 (−1)	12.5 (+1)
10	150 (0)	74.4 (−1.68)	10 (0)
11	150 (0)	225.6 (+1.68)	10 (0)
12	150 (0)	150 (0)	10 (0)
13	105 (−1)	195 (+1)	12.5 (+1)
14	195 (+1)	195 (+1)	12.5 (+1)
15	150 (0)	150 (0)	5.8 (−1.68)
16	150 (0)	150 (0)	14.2 (+1.68)
17	150 (0)	150 (0)	10 (0)

## Data Availability

The data presented in this study are available on request from the corresponding author.

## Supplementary Materials

The following are available online, Figure S1: Response surface as a function of murici pulp and taperabá pulp concentration (A) overall linking attribute (B) taste attribute (C) Texture attribute (D) appearance attribute. Figure S2: Antioxidant potential of murici and tapereba beverages) evaluated by different assays (DPPH, FRAP, ORAC and ABTS).

## Sample Availability

Samples of the compounds are not available from the authors.

## References

[1] Neves L.C., Benedette R.M., Chagas E.A. (2012). Characterization of the Antioxidant Capacity of Natives Fruits From the Brazilian Amazon Region 1. Rev. Bras. Frutic..

[2] Canuto G.B., Xavier A.A.O., Neves L.C., Benassi M.D.E.T. (2010). Physical and Chemical Characterization of Fruit Pulps from Amazonia and Their Correlation To Free Radical Scavenger Activity. Rev. Bras. Frutic..

[3] do Rufino M.S.M., Alves R.E., de Brito E.S., Pérez-Jiménez J., Saura-Calixto F., Mancini-Filho J. (2010). Bioactive compounds and antioxidant capacities of 18 non-traditional tropical fruits from Brazil. Food Chem..

[4] Malta L.G., Tessaro E.P., Eberlin M., Pastore G.M., Liu R.H. (2013). Assessment of antioxidant and antiproliferative activities and the identification of phenolic compounds of exotic Brazilian fruits. Food Res. Int..

[5] Morais L., Maia G.A., Figueiredo R.W. (2011). De Desenvolvimento De Néctares Mistos À Base De Manga E Cajá Enriquecidos Com Frutooligossacarídeos Ou Inulina. Aliment. Nutr..

[6] Alberto P.S., Silva F.G., Cabral J.S.R., De Fátima Sales J., Pereira F.D. (2011). Methods to overcome of the dormancy in murici (Byrsonima verbascifolia Rich) seeds. Semin. Agrar..

[7] Mariutti L.R.B., Rodrigues E., Chisté R.C., Fernandes E., Mercadante A.Z. (2014). The Amazonian fruit Byrsonima crassifolia effectively scavenges reactive oxygen and nitrogen species and protects human erythrocytes against oxidative damage. Food Res. Int..

[8] Gordon A., Jungfer E., Da Silva B.A., Maia J.G.S., Marx F. (2011). Phenolic constituents and antioxidant capacity of four underutilized fruits from the amazon region. J. Agric. Food Chem..

[9] De Souza V.R., Pereira P.A.P., Queiroz F., Borges S.V., De Carneiro J.D.S. (2012). Determination of bioactive compounds, antioxidant activity and chemical composition of Cerrado Brazilian fruits. Food Chem..

[10] Mariutti L.R.B., Rodrigues E., Mercadante A.Z. (2013). Carotenoids from Byrsonima crassifolia: Identification, quantification and in vitro scavenging capacity against peroxyl radicals. J. Food Compos. Anal..

[11] Murillo E., Meléndez-Martínez A.J., Portugal F. (2010). Screening of vegetables and fruits from Panama for rich sources of lutein and zeaxanthin. Food Chem..

[12] Maldonado-Astudillo Y.I., Alia-Tejacal I., Núñez-Colín C.A., Jiménez-Hernández J., Pelayo-Zaldívar C., López-Martínez V., Andrade-Rodríguez M., Bautista-Baños S., Valle-Guadarrama S. (2014). Postharvest physiology and technology of *Spondias purpurea* L. and *S. mombin* L.. Sci. Hortic..

[13] Tiburski J.H., Rosenthal A., Deliza R., de Godoy R.L.O., Pacheco S. (2011). Nutritional properties of yellow mombin (*Spondias mombin* L.) pulp. Food Res. Int..

[14] Prati P., Moretti R.H., Cardello H.M.A.B. (2005). Elaboration of beverage composed by blends of clarified-stabilized sugar cane and juice’s acid fruits. Cienc. Tecnol. Aliment..

[15] Oludemi F.O., Akanbi C.T. (2013). Chemical, antioxidant and sensory properties of tomato-watermelon-pineapple blends, and changes in their total antioxidant capacity during storage. Int. J. Food Sci. Technol..

[16] Neves L.C., Benedette R.M., Tosin J.M., Chagas E.A., Da Silva V.X., Prill M.A.D.S., Roberto S.R. (2011). Production of Blends based on tropical and native fruits from brazilian Amazon. Rev. Bras. Frutic..

[17] Rebouças M.C., Rodrigues M.D.C.P., Afonso M.R.A. (2014). Optimization of the Acceptance of Prebiotic Beverage Made from Cashew Nut Kernels and Passion Fruit Juice. J. Food Sci..

[18] Lawless L.J.R., Threlfall R.T., Meullenet J.F., Howard L.R. (2012). Consumer-based optimization of blackberry, blueberry and concord juice blends. J. Sens. Stud..

[19] Kumar S.B., Ravi R., Saraswathi G. (2010). Optimization of fruit punch using mixture design. J. Food Sci..

[20] Hamacek F.R., Martino H.S.D., Pinheiro-Sant’Ana H.M. (2014). Murici, fruit from the Cerrado of Minas Gerais, Brazil: Physical and physicochemical characteristics, and occurrence and concentration of carotenoids and vitamins. Fruits.

[21] de Mattietto R.A., Lopes A.S., de Menezes H.C.M. (2010). Physical and physicochemical characterization of caja fruit (*Spondias mombin* L.) ans its pulp, obtained using two types of extractor. Braz. J. Food Technol..

[22] Curi P.N., Tavares B.S., Almeida A.B., Pio R., Pasqual M., Peche P.M., de Souza V.R. (2017). Characterization and influence of subtropical persimmon cultivars on juice and jelly characteristics. An. Acad. Bras. Cienc..

[23] Dias F., Abadio B., Galv I., Botelho R. (2012). Physicochemical characteristics and antioxidant activity of three native fruits from brazilian savannah (cerrado) *. Aliment. Nutr..

[24] Rongtong B., Suwonsichon T., Ritthiruangdej P., Kasemsumran S. (2018). Determination of water activity, total soluble solids and moisture, sucrose, glucose and fructose contents in osmotically dehydrated papaya using near-infrared spectroscopy. Agric. Nat. Resour..

[25] Alves A.M., Dias T., Hassimotto N.M.A., Naves M.M.V. (2017). Ascorbic acid and phenolic contents, antioxidant capacity and flavonoids composition of Brazilian savannah native fruits. Food Sci. Technol..

[26] Almeida M.M.B., de Sousa P.H.M., Arriaga Â.M.C., do Prado G.M., Magalhães C.E.D.C., Maia G.A., de Lemos T.L.G. (2011). Bioactive compounds and antioxidant activity of fresh exotic fruits from northeastern Brazil. Food Res. Int..

[27] Zielinski A.A.F., Ávila S., Ito V., Nogueira A., Wosiacki G., Haminiuk C.W.I. (2014). The Association between Chromaticity, Phenolics, Carotenoids, and In Vitro Antioxidant Activity of Frozen Fruit Pulp in Brazil: An Application of Chemometrics. J. Food Sci..

[28] de Souza V.R., Aniceto A., Abreu J.P., Montenegro J., Boquimpani B., de Jesuz V.A., de Campos M.B.E., Marcellini P.S., Freitas-Silva O., Cadena R. (2020). Fruit-based drink sensory, physicochemical, and antioxidant properties in the Amazon region: Murici (*Byrsonima crassifolia* (L.) *Kunth* and *verbascifolia* (L.) DC) and tapereba (*Spondia mombin*). Food Sci. Nutr..

[29] Brunelli L.T., Venturini Filho W.G. (2012). Caracterização Química E Sensorial De Bebida Mista De Soja E Uva *. Aliment. Nutr..

[30] De Carvalho-Silva L.B., Dionísio A.P., Pereira A.C.D.S., Wurlitzer N.J., De Brito E.S., Bataglion G.A., Brasil I.M., Eberlin M.N., Liu R.H. (2014). Antiproliferative, antimutagenic and antioxidant activities of a Brazilian tropical fruit juice. LWT Food Sci. Technol..

[31] Singleton V.L., Orthofer R., Lamuela-Raventós R.M. (1999). Analysis of total phenols and other oxidation substrates and antioxidants by meas of Folin-Ciocalteu reagent. Methods Enzym..

[32] Pękal A., Pyrzynska K. (2014). Evaluation of Aluminium Complexation Reaction for Flavonoid Content Assay. Food Anal. Methods.

[33] Thaipong K., Boonprakob U., Crosby K., Cisneros-Zevallos L., Byrne D.H. (2006). Comparison of ABTS, DPPH, FRAP, and ORAC assays for estimating antioxidant activity from guava fruit extracts. J. Food Compos. Anal..

[34] Brand-Wiliams W., Cuvelier M.E., Berset C. (1995). Use of a free radical method to evaluate antioxidant activity. Food Sci. Technol..

[35] Abreu J., Quintino I., Pascoal G., Postingher B., Cadena R., Teodoro A. (2019). Antioxidant capacity, phenolic compound content and sensory properties of cookies produced from organic grape peel (*Vitis labrusca*) flour. Int. J. Food Sci. Technol..

[36] Prior R.L., Hoang H., Gu L., Wu X., Bacchiocca M., Howard L., Hampsch-Woodill M., Huang D., Ou B., Jacob R. (2003). Assays for hydrophilic and lipophilic antioxidant capacity (oxygen radical absorbance capacity (ORACFL)) of plasma and other biological and food samples. J. Agric. Food Chem..

[37] Freitas D., Mattietto R. (2013). Ideal sweetness of mixed juices from Amazon fruits. Cienc. Tecnol. Aliment..

