# Supplementary figures and images for: Physicochemical Characterization, Antioxidant Capacity, and Sensory Properties of Murici (Byrsonima crassifolia (L.) Kunth) and Taperebá (Spondias mombin L.) Beverages
Source: Molecules. 2021 Jan 11;26(2):332. doi: 10.3390/molecules26020332 (PMC7826852; doi:10.3390/molecules26020332)

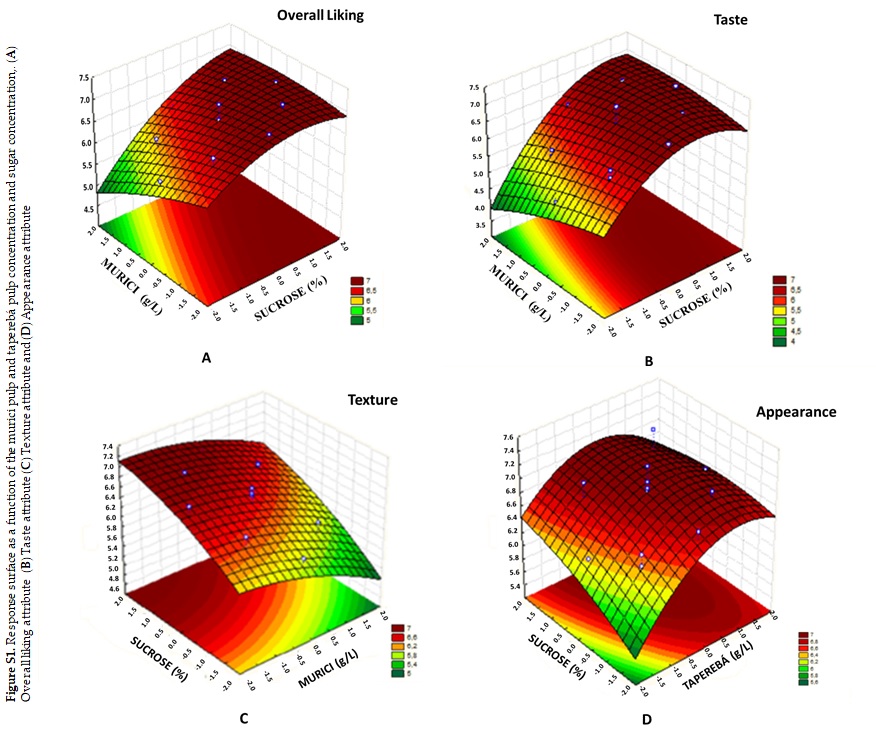

Supplement: Supplementary file 1 [file molecules-26-00332-s001.zip › molecules-1058739-SI.jpg]
